# *CFHTF2* Is Needed for Vegetative Growth, Conidial Morphogenesis and the Osmotic Stress Response in the Tea Plant Anthracnose (*Colletotrichum fructicola*)

**DOI:** 10.3390/genes14122235

**Published:** 2023-12-18

**Authors:** Chengkang Zhang, Ziwen Zhou, Tianlong Guo, Xin Huang, Chengbin Peng, Zhideng Lin, Meixia Chen, Wei Liu

**Affiliations:** 1 Industry and University Research Cooperation Demonstration Base of Science and Technology Agency in Fujian Province, College of Life Science, Ningde Normal University, Ningde 352100, China; chengkangzhang@126.com (C.Z.); zzw1174600720@163.com (Z.Z.); tlongguo@163.com (T.G.); 15060131660@163.com (X.H.); pengcb625@163.com (C.P.); t2125@ndnu.edu.cn (Z.L.); cmx_101019@163.com (M.C.); 2 Key Laboratory of Bio-Pesticide and Chemistry Biology, Fujian Agricultural and Forestry University, Ministry of Education, Fuzhou 350002, China; 3 College of Food Science, Fujian Agriculture and Forestry University, Fuzhou 350002, China; 4 College of Life Science, Fujian Agriculture and Forestry University, Fuzhou 350002, China

**Keywords:** tea plant, *C. fructicola*, *CfHTF2*, RNA-seq

## Abstract

Tea is an important cash crop worldwide, and its nutritional value has led to its high economic benefits. Tea anthracnose is a common disease of tea plants that seriously affects food safety and yield and has a far-reaching impact on the sustainable development of the tea industry. In this study, phenotypic analysis and pathogenicity analysis were performed on knockout and complement strains of *HTF2*—the transcriptional regulator of tea anthracnose homeobox—and the pathogenic mechanism of these strains was explored via RNA-seq. The *MoHox1* gene sequence of the rice blast fungus was indexed, and *the anthracnose genome* was searched for *CfHTF2*. Evolutionary analysis recently reported the affinity of HTF2 for *C. fructicola* and *C. higginsianum*. The loss of *CfHTF2* slowed the vegetative growth and spore-producing capacity of *C. fructicola* and weakened its resistance and pathogenesis to adverse conditions. The transcriptome sequencing of wild-type N425 and *CfHTF2* deletion mutants was performed, and a total of 3144 differentially expressed genes (DEGs) were obtained, 1594 of which were upregulated and 1550 of which were downregulated. GO and KEGG enrichment analyses of DEGs mainly focused on signaling pathways such as the biosynthesis of secondary metabolites. In conclusion, this study lays a foundation for further study of the pathogenic mechanism of tea anthracnose and provides a molecular basis for the analysis of the pathogenic molecular mechanism of *CfHTF2*.

## 1. Introduction

The tea plant (*Camellia sinensis* (L.) O. Kuntze) is native to China and has been consumed for more than 5000 years [1]. Fujian Province is an important tea-producing province in China, and the tea industry is an important characteristic industry of modern agriculture in the province. Tea gardens are distributed from coastal flatlands to hilly mountains, and the climate and environment of each region are different. Tea varieties have a strong ability to adapt to the environment [2,3]. Tea is one of the world’s three major drinks, and daily tea consumption has become a common habit. Today, with the increase in people’s demand for tea, tea garden planting areas with production demand have also expanded, but the construction and management of tea gardens are imperfect, only single varieties are planted, and tea garden species diversity is relatively minimal, resulting in the long-term persistence of tea plant diseases [4]. At present, different tea plant diseases worldwide have had different degrees of impact on tea farmers, and there are more than 500 different tea plant diseases worldwide [4]. There are four types of anthrax diseases caused by anthracnose fungi: anthracnose is a common tea plant leaf disease in which dark leaf lesions occur, and severe disease outbreaks inflict economic losses, causing many leaves to fall off and the whole plant to die [5,6].

*Colletotrichum* spp. are considered one of the ten most important pathogenic fungi in the world [5,6]. Distributed worldwide, the fungi of this genus can harm a variety of crops, such as cereals, tea and fruits, causing severe economic losses in agricultural and forestry production [7]. Tea plant anthracnose disease is common in tea areas across the world, and scholars have studied anthracnose-infected leaves in eight tea-producing countries worldwide for detailed research and five pathogenic fungi, namely, *C. camelliae*, *C. boninense*, *C. gloeosporioides*, *C. fructicola* and *C. crassipes* [8,9]. In recent years, in summer and autumn, the weather has been hot, and there has been more rainfall, resulting in high humidity in the tea garden environment, which is very suitable for anthracnose germination growth [7,8,9]. When anthracnose conidiacans are attached to tea under inhospitable conditions, the long incubation period of spores can reach several months prior to disease onset. When the temperature is suitable, the attached cells invade from the pores on the back of the leaves, gradually forming brown or gray-white disease spot tissue from black dots. The diseased spots become brittle, and anthracnose can invade the young shoots of tea plant leaves [7,8,9]. Disease spots gradually expand as the leaves grow, and the disease spots visible to the naked eye are actually conidial discs [10]. Conidiacan spores use their sporangia pressure alongside wind, rain, pests and other large-scale spreaders to reach tea plants and remain latent, waiting for an opportunity to infect the plant and making complete removal difficult [11]. Anthracnose can invade on its own, but can also use open leaf and branch wounds, especially after harvesting. The occurrence of new trauma directly provides favorable conditions for anthracnose infection [12]. Therefore, tea plant anthrax seriously threatens the development of the tea industry. Tea plant anthracnose is difficult to completely eradicate, and its latent and infectious characteristics cause economic losses in the tea industry.

The homeobox proteins encoded by the homeobox gene family are a class of transcriptional regulators that are structurally and functionally highly conserved [13]. Garber et al. reported that mutations in the Antp gene in fruit flies can change their original expected growth and development pathways [14]. In the model strain *Saccharomyces cerevisiae*, there are two negative regulatory homeobox gene transcription factors, *Yhp1* and *Yox1p*, which affect the physiological activity of M/G1 during cell meiosis in sexually reproducing organisms [15,16]. *MoHox2*, a member of the homeobox family of the rice blast fungus (*Magnaporthe oryzae*), specifically regulates the formation of the conidia of the rice blast fungus and the morphological development of the conidia at the tip of the spore stalk [17,18]. *MoHox1* mainly regulates the vegetative growth of hyphae, resulting in colony deformity and hyphal densification [17]. In *Fusarium graminus*, *HTF1* and *HTF8* regulate hyphal growth and conidial formation during the growth and development of *Fusarium graminearum*, while other members of the homeobox family are not associated with pathogenicity [19,20]. The homeobox gene *GRF10* in *Candida albicans* plays an important role in regulating mycelial growth, virulence and spore and biofilm formation [21]. In view of the study of the homeobox gene in other fungi, which may also be involved in the growth and development of pathogenic fungi and pathogenic infection in tea plant anthracnose pathogens, no reports of homeobox gene function have been found in tea plant anthracnose. Therefore, this study performed a systematic evolutionary analysis of *CfHTF2* and investigated the effects of *CfHTF2* on the vegetative growth, spore-producing capacity, resistance and pathogenicity of tea plant anthracnose bacteria by knocking out *CfHTF2* (Δ*Cfhtf2-178*). Through RNA-seq, the molecular mechanism of *CfHTF2* pathogenesis was preliminarily resolved. This paper lays a foundation for an in-depth study of the pathogenic mechanism of tea plant anthracnose and provides a molecular basis for the analysis of the pathogenic molecular mechanism of *CfHTF2*.

## 2. Materials and Methods

### 2.1. Determination of Pathogenicity

The Koch-style rule was used to verify the pathogen, and the tea plant pathogen was connected with a 5 mm hole punch. The sterilization cake and the blank agar plate were connected to the ex vivo tea leaf back-infestation. The adult leaves of Fuyun No. 6 tea plants were completely immersed in 75% alcohol, soaked for 1 min, and washed with sterile water 3 times. After gently scratching the left and right sides of the leaf (without piercing) with an inoculation needle, the fungus cake was inserted into the wound, after which the plants were symmetrically inoculated. The leaves of the isolated and purified fungal strains were inoculated as the experimental group, the leaves of the blank media were used as the control group, the petiole of the tea plant leaf was wrapped with wet cotton, and the fungus cake was covered with cotton. The mixture was incubated at 26 °C. The leaf symptoms were observed daily, and images were taken.

### 2.2. Generation of Cfhtf2 Deletion Mutants and Complementary Strains

To generate *CfHTF2* deletion fragments, we first used the primer pairs Htf2AF/Htf2AR and Htf2BF/Htf2BR to amplify the upstream and downstream fragments of the *CfHTF2* gene from the genome of *C. fructicola* wild-type strain N425, and the resulting amplicons were infused with different parts of the geneticin resistance gene by overlapping PCR (Table S1). Protoplasting buffer (0.8 g of lysing enzymes (L1412; Sigma-Aldrich, Inc., Shanghai, China) and 20 mL of 1 M KCl solution) was used for protoplast preparation of *C. fructicola*. The details of the protoplast preparation and fungal transformation are described in an established protocol [22]. Geneticin-resistant transformants were screened via PCR with the primer pair Htf2OF/Htf2OR (Table S1) and further verified via Southern blotting.

For complementation of the *CfHTF2* deletion, first, a fragment containing the *CfHTF2* ORF and its native promoter was amplified via the primer pair Htf2CF/Htf2CR (Table S1). Second, this fragment was cloned and inserted into the pDL2 plasmid, which carries a hygromycin resistance gene. Third, the resulting vector harboring the CfHTF2 ORF integrated with the GFP fragment was transformed into the protoplast of the *CfHTF2* deletion mutant (Δ*Cfhtf2-B178*). Hygromycin-resistant transformants were screened via PCR and fluorescence signal detection.

### 2.3. Vegetative Growth of Colonies

On PDA media, anthracnose wild-type N425 and the transformants were incubated at 26 °C for 7 days, aerial hyphae on the surface were scraped off with a sterile knife, and 5 mm of fungal material was removed from the edge of the colony, placed on different media plates (potato glucose agar (PDA), complete medium (CM), oat medium (OA) and rice bran medium (RBM)) at 9 cm with a sterile pick and needle and incubated in a light incubator at 26 °C for 5 days. The colony diameters of the wild-type and transformant strains were measured via the cross method and photographed. Three biological replicates were set up per experiment.

### 2.4. Spore Yield, Conidiation and Appressorium Observation

Spore yield: On PDA media, tea plant anthracnose wild-type N425 were cultured at 26 °C for 10 days, the spores were eluted on the surface of PDA plates, the hyphae were filtered with a Miracloth membrane to obtain a spore–liquid volume of 2 mL, 100 µL of spore stock solution was diluted 10 times, and 10 µL was drawn to calculate the plate statistics with blood cells and calculate spore production. The conidia of each strain were observed under a microscope and photographed. For the appressorium formation assay, conidia were resuspended in sterile distilled water to 5 × 10^4^ conidia mL^−1^. Drops of 50 mL of conidium suspension were placed on glass coverslips (Fisher Scientific, Pittsburgh, PA, USA) and incubated in a moist chamber at room temperature.

### 2.5. Sensitivity to Stress Factors

To prepare solid stress media, 5 mm mold cake was taken from the edge of the colony and inoculated in different stress media (1 M of sodium chloride (NaCl), 1 M of potassium chloride (KCl), 100 μg/mL of fluorescent protein whitening agent (CFW), 0.01% sodium lauryl sulfate (SDS), 0.03% Congo red (CR), 0.05% hydrogen peroxide (H_2_O_2_) and 1 M of sorbitol (sorbitol)), which were added to the CM. Agar powder (15 g/L, natural pH = 121 °C) was autoclaved. Center images were taken after incubation at 26 °C in the dark for 5 days.

### 2.6. RNA-Seq Analysis

RNA was extracted using the Eastep® Super Total RNA Extraction Kit, and RNA integrity was detected via 1% agarose gel electrophoresis. The RNA was stored in a −80 °C freezer, the extracted total RNA was transported to Nuowo (Beijing, China) for sequencing using dry ice, and the extracted RNA was fragmented using PCR plates with magnetic plate holders. The fragmented mRNA was reverse-transcribed to cDNA using superscript II and random primers (Invitrogen, Carlsbad, CA, USA).

Data filtering and quality control were carried out with fastp (Version: 0.23.4) software, and the resulting clean data were used for subsequent analysis [23]. StringTie was used for read assembly quantification. The fragments per kilobase of exon per million fragments mapped (FPKM) value refers to the number of reads per thousand bases of exons per million maps, and the amount of gene expression was calculated using the FPKM method [24,25]. DESeq2 was used to calculate the fold change in expression of genes between different samples according to the expression level of the genes [26]. A *p*-value < 0.05 and a median|log2-fold change| ≥ 1 were used as the standards for screening differentially expressed genes. GO and KEGG enrichment analyses were performed on all DEGs [27].

### 2.7. qRT-PCR

Primer (Version: 5.0) software was used to design primers, with universal tubulin serving as the internal reference gene. The reaction solutions were prepared separately for qRT-PCR validation, and 3 replicate qRT-PCR analyses were performed using Roche LC480 equipment (Roche Diagnostics GmbH Mannheim, Mannheim, Germany) and SYBR Green (Takara Bio, Inc., Shiga, japan). We initially used a two-step PCR amplification procedure with predenaturation at 95 °C for 30 s, followed by 40 cycles of denaturing at 95 °C for 5 s and annealing at 60 °C for 34 s. Amplification, dissolution and standard curves were automatically generated via Roche LC480 (Version: 2.2) software. GeNorm (Version: 2.2) software was used to calculate the relative expression levels of the genes of interest.

## 3. Results

### 3.1. CfHTF2 Evolutionary Analysis and Acquisition of Knockout Mutants and Complementary Strains

**Figure 1:**
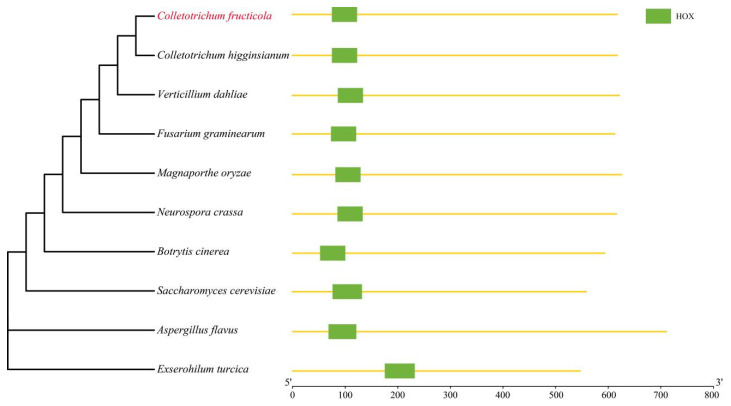
Phylogenetic trees and conserved domains of HTF2 in different species.

*MoHox1* (*M. oryzae*) was named with the rice blast fungus *M. oryzae HOX1* gene MGG 04853 (the gene sequence of *M. oryzae* 70-15), and the HTF2 sequence (named *CfHTF2*) was searched for in the *C. fructicola* genome. To further analyze the kinship of *CfHTF2* between different species, the selected HTF2 protein sequences of *Magnaporthe oryzae* (GenBank: XP_003712331.1), *Verticillium dahliae* (GenBank: KAF3347114.1), *Fusarium graminearum* (GenBank: XP 011328782.1), *Exserohilum turcica* (GenBank: XP008029609.1), *Aspergillus flavus* (GenBank: AFLA 069100), *Neurospora crassa* (GenBank: XP_961320.1), *Botrytis cinerea* (GenBank: EMR85177), *Colletotrichum higginsianum* (GenBank: XP_018160237.1) and *Saccharomyces cerevisiae* (GenBank: AJV10273) were selected to construct phylogenetic trees and conserved domain models (Figure 1). The HTF2 proteins of *C. fructicola* and *C. higginsianum* were clustered in a single clade, with the closest affinity and 99% similarity, and the two species were also the closest relatives, indicating that the results of the phylogenetic tree construction were reliable. Domain analysis of each species revealed that HTF2 contained a HOX domain near the 5’ end, and the closer the evolutionary tree was to each other, the closer the location of the domain was (Figure 1).

**Figure 2:**
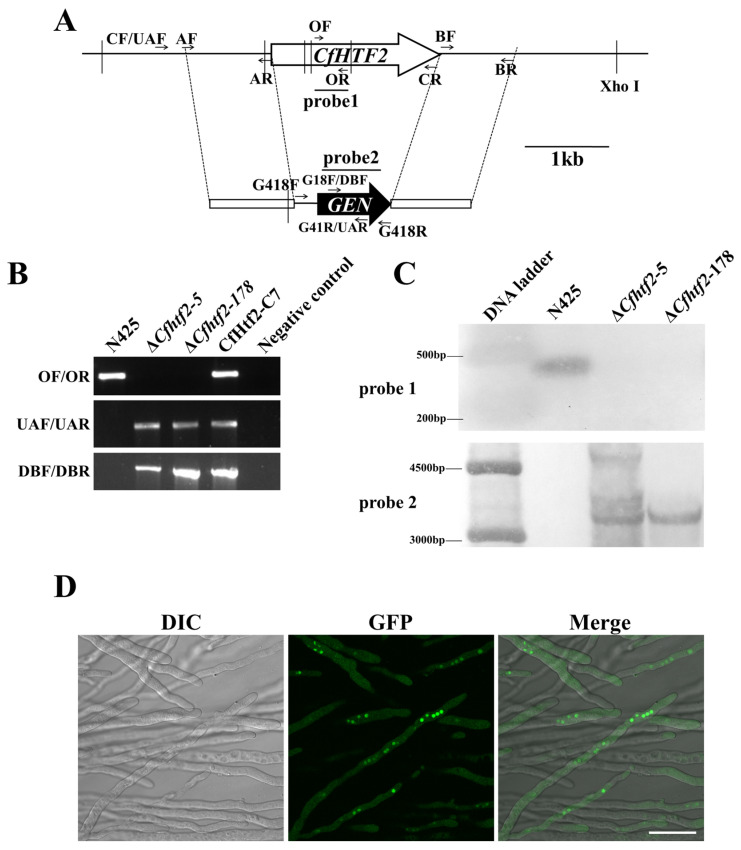
(**A**) Schematic diagram of the *CfHTF2* gene knockout, (**B**) knockout mutant PCR detection, (**C**) Southern blot analysis of candidate knockout transformants of genes involved in DNA methylation, (**D**) fluorescent observation of the CfHtf2-C7 strains.

To analyze the biological function of the *CfHTF2* gene in *C. fructicola*, the isolated strain N425 was used as a wild type, and *CfHTF2* was knocked out via homologous recombination. The corresponding *CfHTF2* knockout transformant was obtained, and a preliminary screening was carried out via PCR. The screening results were analyzed via Southern blot, and one positive knockout mutant was named Δ*Cfhtf2-178* (Figure 2A–C). To further explore the function of the gene of interest in the growth and development of *C. fructicola*, HTF2, which contains its own promoter and was fused with the GFP gene, was transferred into the protoplast of Δ*Cfhtf2-178* to obtain complementary transformants. As determined via fluorescence microscopy, the fluorescence signal was mainly localized in the nucleus, further indicating that *CfHTF2* is mainly localized in the nucleus (Figure 2D). The complementary strain was again verified via PCR, and the true positive complementary transformant CfHtf2-C7 (Figure 2) was obtained through hygromycin resistance plate screening and fluorescence signal detection.

### 3.2. Loss of CfHTF2 Slows the Vegetative Growth of C. fructicola

**Figure 3:**
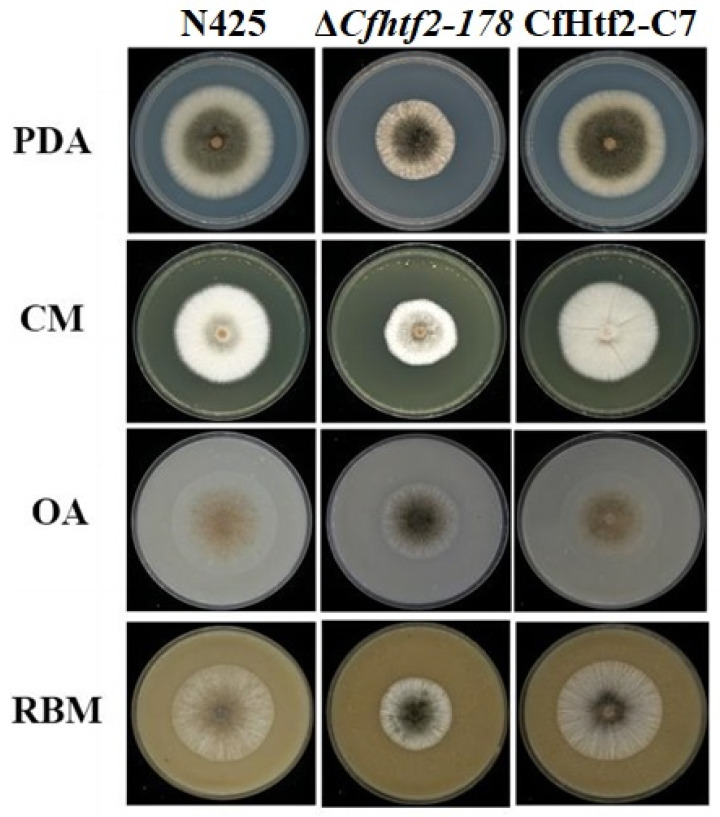
Morphological characteristics of N425, Δ*Cfhtf2-178* and CfHtf2-C7 colonies on different media. The culture phenotype was cultured for 5 days.

To analyze the function of *CfHTF2* in regulating vegetative growth processes, wild-type N425, Δ*Cfhtf2-178* and CfHtf2-C7 were inoculated into PDA, CM, OA or RBM media and cultured for 5 days in a light incubator at 26 °C for 12 h/12 h. Compared with wild-type N425 and CfHtf2-C7, Δ*Cfhtf2-178* significantly decreased the colony growth diameter on different media, and Δ*Cfhtf2-178* became denser in terms of colony morphology (Figure 3). There was no significant difference in colony morphology or colony diameter between the wild-type and CfHtf2-C7 plants (Figure 3). These findings indicate that the loss of *CfHTF2* slows the vegetative growth of *C. fructicola* and that *CfHTF2* is involved in the regulation of *C. fructicola* hyphal growth.

### 3.3. Effect of The CHTF2 Gene on Asexual Reproduction in Anthracnose

**Figure 4:**
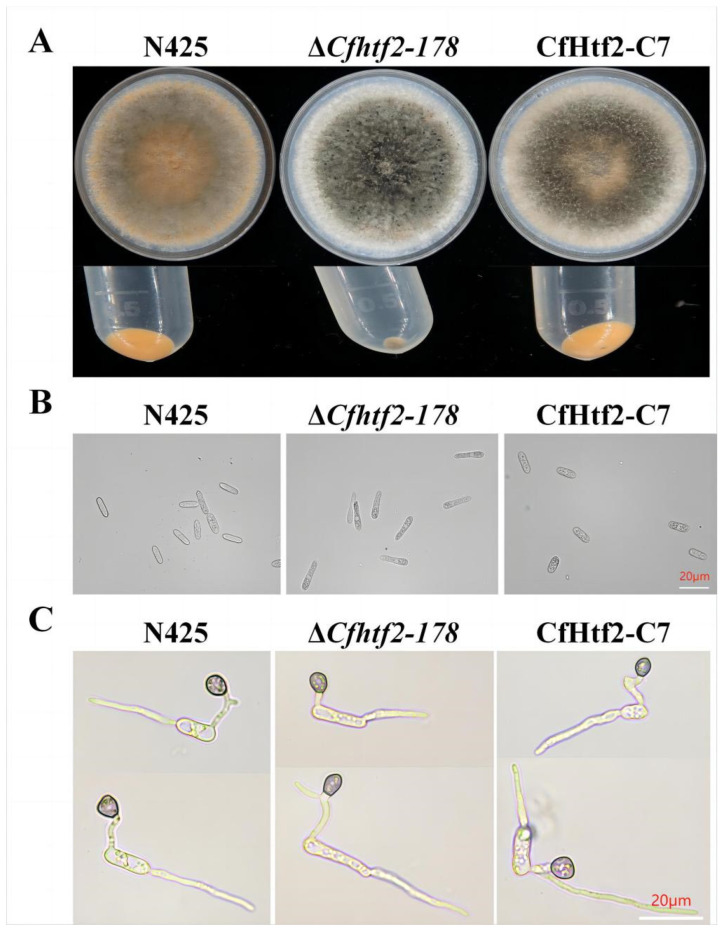
Spore production and conidia of N425, Δ*Cfhtf2-178* and CfHtf2-C7. (**A**) Conidia produced on the surface of the medium; (**B**) conidial structure; (**C**) attached spore structure. The culture phenotype was cultured for 10 days.

Anthracnose infection mainly relies on conidial transmission and germination, and the fungus gradually becomes the central disease strain. Therefore, to explore whether CfHTF2 affects the asexual reproduction of tea plant anthracnose bacteria, tea plant anthracnose wild-type N425, Δ*Cfhtf2-178* and CfHtf2-C7 plants were inoculated on PDA media, placed in a light incubator at 26 °C for 10 days and eluted with sterile water and filtered hyphae, after which the volume of the spore solution was adjusted to 2 mL. Under the same conditions, there were obvious differences in the yields of wild-type N425, Δ*Cfhtf2-178* and CfHtf2-C7 spores on PDA media, and the wild-type and CfHtf2-C7 orange spores were distributed throughout the whole dish (Figure 4A). After 5 min of centrifugation at 8000 rpm, the Δ*Cfhtf2-178* centrifuge tube contained the least amount of spores (Figure 4A). The results of colony conidia further showed that all three strains could produce conidia, and the number of conidia on the conidia of Δ*Cfhtf2-178* decreased (Figure 4A). The morphology of the Δ*Cfhtf2-178* conidia changed, the conidia became more elongated and the complementary strains returned to normal. Although the loss of HTF2 caused abnormal conidial morphology, it did not affect the formation of attachment cells (Figure 4B,C). This result shows that the loss of *CfHTF2* affected the spore production capacity of *C. fructicola* and played an important role in the process of asexual reproduction.

### 3.4. Response of CfHTF2 to Anthrax under Different Stresses

**Figure 5:**
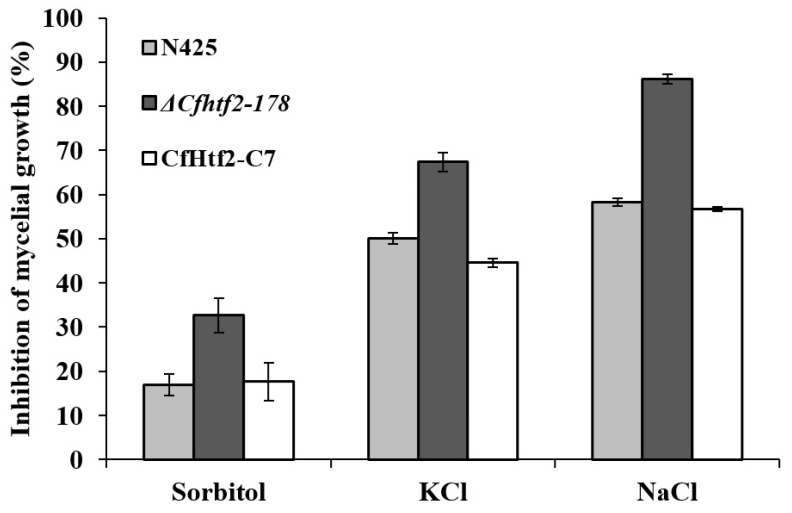
Statistical analysis of cell-sensitive colonies of wild-type N425, Δ*Cfhtf2-178* and CfHtf2-C7 tea anthracnose plants under stress (sorbitol, sodium chloride and potassium chloride).

The fungal cell wall plays an important role in maintaining cell hyphal morphology and coping with various adverse external environments during fungal growth and development. To further study the response mechanism of *CfHTF2* to tea plant anthracnose under different stresses, tea plant anthracnose wild-type N425, Δ*Cfhtf2-178* and CfHtf2-C7 plants were inoculated on CM media supplemented with 1 M of NaCl, 0.03% CR, 1 M of KCl, 1 M of sorbitol, 100 μg/mL of CFW, 0.01% SDS, and 0.05% H_2_O_2_, with empty CM media serving as the control (Figure 5). The inhibition rates of CFW, CR, SDS and H_2_O_2_ media on the Δ*Cfhtf2-178* strains were the same as those on the wild-type and CfHtf2-C7 strains, and there was no difference. However, treatment with sorbitol, KCl or NaCl had a greater inhibitory effect on the knockout mutants than on the wild-type plants, indicating that the knockout plants were more sensitive to osmotic stress than the wild-type plants (Figure 5). These results suggest that the loss of CfHTF2 decreases the hyphal growth tolerance of *C. fructicola* under high osmotic pressure conditions.

### 3.5. Determination of Pathogenicity

**Figure 6:**
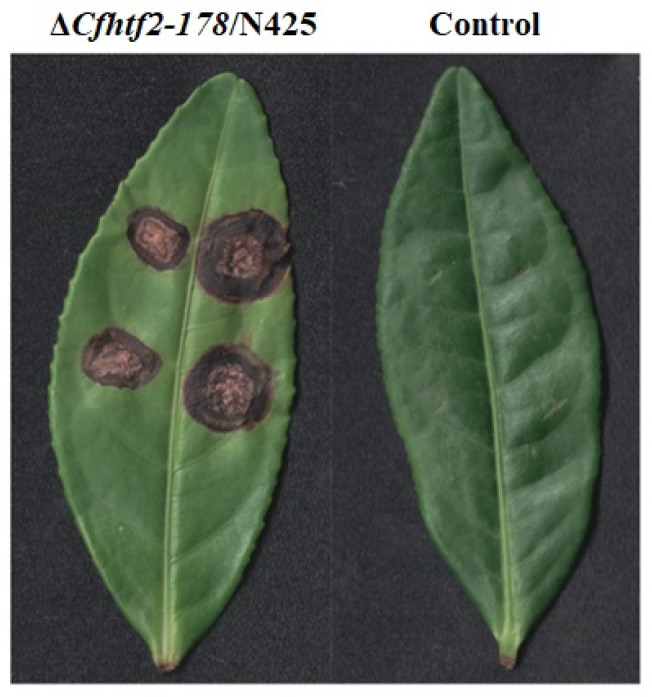
Pathogenicity of N425 and Δ*Cfhtf2-178*.

To explore whether HTF2 deficiency affects tea plant anthracnose infection, 5 mm long fungus cakes were taken from the colony edge of the N425 and Δ*Cfhtf2-178* strains, inoculated on the leaves of Fuyun No. 6 tea plants in vivo and placed in light and dark alternating moisturizing media at 26 °C (Figure 6). On the 5th day, the leaves produced disease spots, the wild-type disease spots were larger than the mutant disease spots and no disease spots were observed in the blank control group. (Figure 6). The results showed that the pathogenicity of CfHTF2 was weakened due to the loss of CfHTF2, which was presumably caused by the slowdown of vegetative growth in the colonies.

### 3.6. RNA-Seq Analysis

**Figure 7:**
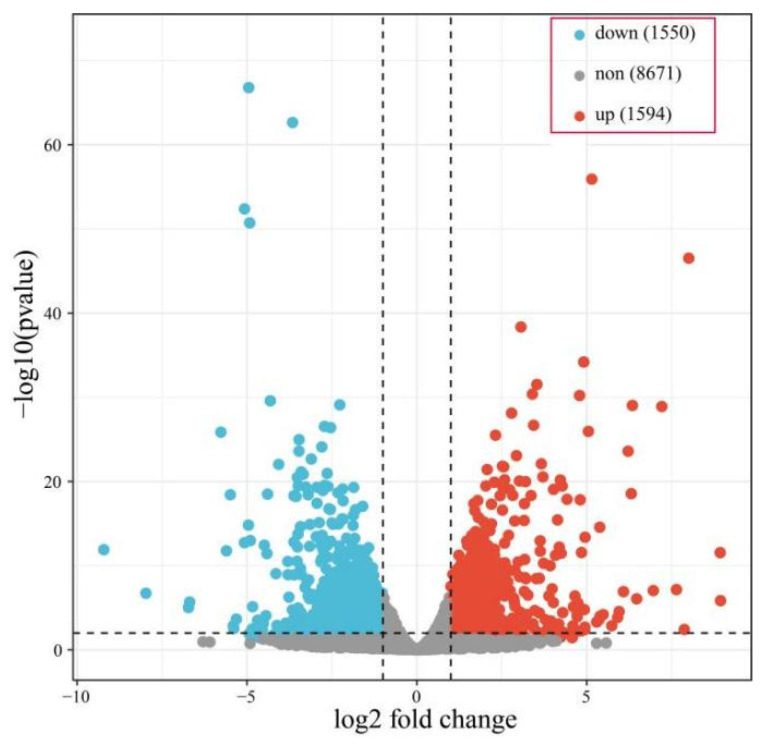
The number of differentially expressed genes in N425 and Δ*Cfhtf2-178*.

Three biological replicates of RNA-seq were performed on the wild-type N425 and Δ*Cfhtf2-178 strains*, resulting in a total of 39.03 Gb of clean data. The between-sample correlation and PCA showed that the interreplicate correlation exceeded 0.95, and the replicates were aggregated together. These results indicate that the RNA-Seq data are reliable and suitable for further analysis. Eight genes were randomly selected for three independent replicates of qRT-PCR analysis, and the transcriptome data were significantly correlated with the qRT-PCR data (R2 = 0.91; Figure S1). The results show that the test sampling was reasonable and that the RNA-seq data quality was reliable. First, differential expression analysis was carried out, and a total of 3144 DEGs were found—1594 upregulated and 1550 downregulated ones—between N425 and Δ*Cfhtf2-178* (Figure 7).

### 3.7. GO and KEGG Enrichment Analysis

**Figure 8:**
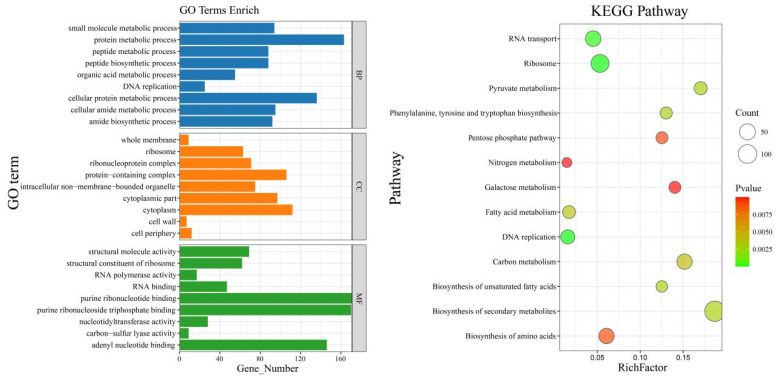
GO and KEGG enrichment analyses of the DEGs.

First, Gene Ontology (GO) enrichment analysis was performed on 3144 DEGs, mainly focusing on the amide biosynthetic process, peptide biosynthetic process, cellular amide metabolic process, peptide metabolic process, DNA replication, cellular protein metabolic process, small molecule metabolic process, protein metabolic process and organic acid metabolic process (Figure 8). The DEGs identified via KEGG analysis were involved in the biosynthesis of secondary metabolites, ribosomes, DNA replication, RNA transport, pyruvate metabolism, the biosynthesis of unsaturated fatty acids, phenylalanine, tyrosine and tryptophan biosynthesis, fatty acid metabolism, carbon metabolism, the pentose phosphate pathway, the biosynthesis of amino acids, galactose metabolism and nitrogen metabolism (Figure 8). The biosynthesis of secondary metabolites was determined via the enrichment of downregulated genes, and these pathways have a certain impact on the metabolism and transport of substances during mycelial life activities, thereby regulating the growth of hyphae. This may also lead to a decrease in the secretion of toxins during infection of the host, which may be related to the weakening of mutant pathogenicity. Further analysis revealed that the downregulated genes were associated mainly with carbohydrate enzymes (*A03859* and *A05202*) and ubiquitin ligase chips (*A07575*), and it was reported that carbohydrate enzymes can degrade the cell wall and help invade host cells.

## 4. Discussion

Research on the gene function and pathogenic mechanism of tea plant anthracnose has become increasingly extensive and in-depth, providing an important theoretical basis for cultivating disease-resistant tea plant varieties, developing low-toxicity and environmentally friendly pesticides and effectively controlling the spread of tea plant anthracnose bacteria, which is conducive to promoting the selection and breeding of high-quality tea plant varieties and ensuring tea safety [28,29,30]. The loss of *CfHTF2* had a significant effect on the vegetative growth of the tea plant anthracnose fungus *C. fructicola*, the vegetative growth of the mutants was significantly slower than that of the wild type and the colony morphology of the mutants was denser than that of the wild type. The deletion of the homologous box gene *GRF10* in *Candida albicans* led to the inhibition of hyphal growth, defective spore and biofilm formation and reduced virulence in mice. The discovery of a loss of HTF1 in *F. graminearum* resulted in defects in the development of *F. graminearum* and the inhibition of its conidial synthesis. Htf1 (HOX2), reported in *M. oryzae*, plays a key role in the spore development of rice blast bacteria, and the loss of Mohtf1 causes rice blast bacteria to lose the ability to form conidia. The loss of *CfHTF2* caused the spore morphology to change, and the spores became rod-shaped spores; moreover, it was speculated that *CfHTF2* may participate in the regulation of homeostasis inside cells, resulting in the malformation of the spore morphology. Through the sensitivity of cells to eight stress factors, it was further proven that the loss of *CfHTF2* increased significantly under high-osmolality conditions, and it was speculated that due to the loss of *CfHTF2*, the osmotic pressure of cells under spore morphology decreased, and malformation occurred. However, there is no direct evidence that intracellular osmolality and the transcription factor *CfHTF2* intersect, and further research is needed.

In terms of the pathogenicity of infected tea, the production of tea disease spots relies mainly on the spores and hyphae of anthracnose to infect tea cells [5,6,7,8]. Before and after the deletion of the *CfHTF2* gene, the spore production capacity changes, and the production of conidia decreases, which does not affect the germination of spores or the formation of appressoria; these spores mechanically penetrate the process of infecting tea plant leaf cells and are not damaged due to the loss of *CfHTF2*. The results from the infection of tea plant leaves showed that the disease spots of the mutants were slightly smaller than those of the other strains, and it was speculated that the pathogenicity was weakened due to the loss of *CfHTF2*, the slower growth of nutrients and the change in spore morphology. The HTF2 genes of the rice blast species *MoHOX3*, *Fusarium graminearum FgHTF8* and *Aspergillus flavus AfHTF2* are homologous proteins, and the HTF2 studies of the above strains have shown consistent results. HTF2 is highly conserved in different species, and its functional vegetative growth is related to asexual reproduction [17,19,31].

Through transcription sequencing analysis, it was found that the signaling pathway regulated by *CfHTF2* is extremely complex. *CfHTF2* deletion had different degrees of influence on various secondary metabolite anabolic pathways, the cell cycle, protein synthesis translation transport, ribosome transport, etc., thus possibly affecting the growth of *C. fructicola* by affecting biosynthesis and material metabolism transport.

The differentially expressed genes involved in the synthesis of secondary metabolites, carbohydrate enzymes and a secondary metabolite gene were screened, and studies have reported that carbohydrate enzyme CAZymes play an important role in infecting plant cells [32,33,34]. *CfHTF2* may regulate CAZy enzymes and secondary metabolic pathways. In *Aspergillus*, HTF also regulates toxins in this metabolic pathway. In addition, CAZy enzymes and secondary metabolism are related to cell wall formation and pathogenicity, and HTF may, therefore, indirectly affect pathogenicity to a certain extent [32,33,34]. The transcription of genes associated with the synthesis of these secondary metabolites decreased. After knocking out *AfHTF2* in *Aspergillus flavus* [35]. The synthesis of *Aspergillus flavus* was completely inhibited, and the resulting decrease in secondary metabolism synthesis by *AfHTF2* was confirmed via the transcriptional sequencing results of the tea plant anthracnose HTF2 [35]. It is speculated that HTF2 may be used as a global regulator of secondary metabolites, further indicating that different species of HTF2 play important functional roles.

## 5. Conclusions

In this study, through a sequence analysis of the *CfHTF2* gene of the homeobox family of *C. fructicola*, a tea plant anthracnose fungus, it was found that the HTF2 gene has a HOX protein structure that is highly conserved among different species. Phenotypic analysis of knockout mutants and replenishing strains revealed that *CfHTF2* could affect the vegetative growth and conidiation of *C. fructicola*, which was significantly inhibited under different osmotic-pressure-related stresses. However, the pathogenicity of these viruses in tea plants is weakened. A total of 3144 DEGs were obtained based on RNA-Seq data, 1594 of which were upregulated and 1550 of which were downregulated. Enrichment analysis revealed that the genes were associated mainly with the cell cycle, protein synthesis translation, ribosome transport, secondary metabolite synthesis and mismatch repair. These results indicate that *CfHTF2* plays a crucial role in anthracnose in tea plants, laying a molecular basis for an in-depth analysis of its molecular mechanism of pathogenesis.

## Supplementary Materials

The following supporting information can be downloaded at: https://www.mdpi.com/article/10.3390/genes14122235/s1, Figure S1: Scatter plot of the correlation between the gene expression levels from the transcriptome and qRT-PCR data; Table S1: All primers used in this study.
