# Supplementary figures and images for: CFHTF2 Is Needed for Vegetative Growth, Conidial Morphogenesis and the Osmotic Stress Response in the Tea Plant Anthracnose (Colletotrichum fructicola)
Source: Genes (Basel). 2023 Dec 18;14(12):2235. doi: 10.3390/genes14122235 (PMC10743015; doi:10.3390/genes14122235)

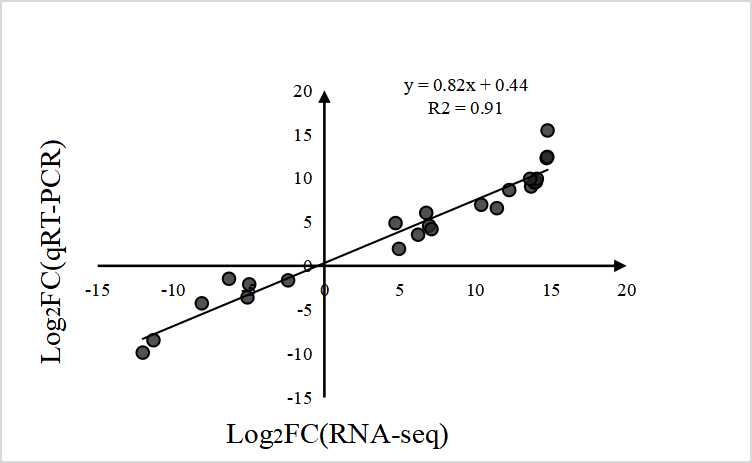

Supplement: Supplementary file 1 [file genes-14-02235-s001.zip › Figure S1.jpg]
